# The complete mitochondrial genome of the Qinghai Tibetan pig

**DOI:** 10.1080/23802359.2018.1502631

**Published:** 2018-08-17

**Authors:** Qian-Yun Ge, Yuan Cai, Guo-Shun Chen, Tian-Tuan Jiang, Qiao-Li Yang, Xiao-Yu Huang, Sheng-Guo Zhao

**Affiliations:** College Of Animal Science And Technology, Gansu Agricultural University. No. 1 Yingmen Village, Anning District, Gansu Province, China

**Keywords:** Qinghai Tibetan pig, mitochondrial DNA, NJ phylogenetic tree

## Abstract

The complete mitochondrial genome sequence of the Qinghai Tibetan pig was first determined in this study. The total length of the mitogenome is 16,720 bp. Indicating the an A + T(60.5%)-rich feature, including 2 ribosomal RNA genes, 13 protein-coding genes. 22 transfer RNA genes and 1 non-coding control region. The NJ phylogenetic tree analysis showed that the phylogenetic relationship between Qinghai Tibetan pig and Yimenghei pig was the closest, and the relationship with Chinese northeas wildboar was farthest.

Qinghai Tibetan pig allowed them to adapt to extreme conditions such as hypoxia differences with low-altitude pigs (Mingzhou Li et al., 2013). This study first reported complete mitochondrial DNA sequence of Qinghai Tibetan pig and compared with other breeds. The sample was farmed at the Yushu city of Qinghai province in China (Altitude 4493 meter, East longitude 97^a^, Northern latitude 33^a^) and stored in the laboratory of Gansu Agricultural University. Total genomic DNA was extracted using the EasyPure Kit of Genomic DNA (Transgen Biotech, Beijing, China). PCR was carried out with 24 pairs of primers designed according to the Landrace pig (GenBank accession number NC_000845.1) (Dong Xu et al., 2013). DNA sequence was analyzed using MEGA7 software (Kumar et al., 2016).

The total length of the mitogenome is 16,720 bp. With the base composition of 34.7% A, 25.8% T, 26.2% C, 13.3% G and an A + T (60.5%)-rich feature. Including 2 ribosomal RNA genes,13 protein-coding genes, 22 transfer RNA genes and 1 non-coding control region (D-loop region). The arrangement of these genes was the same as that found in the Landrace pig. Besides the ND2, ND3 and ND5 initiation codon is ATA, ND4L is GTG, ND6 is TTA and the rest of the proteins are ATG. All the mitogenome genes were encoded on the L strand except for three tRNA genes (tRNA^lle^, tRNA^Asp^ and tRNA^Leu^). Of all these genes were found 7 overlaps and 12 spaces in the length of 1-43 bp. These genes had five types of termination codon, including CAT for ND1 and ND6, ACT for ND2, TAA for COX1, ATP8, ATP6, ND4L, ND5, AGA for Cytb, and an incomplete termination codon T- for COX2, COX3, ND3, ND4. The length of D-loop region was 1284 bp. A phylogenetic tree constructed from the complete mtDNA genomes of Qinghai Tibetan pig and 26 Chinese and 7 European animals is shown in Figure 1. The analysis found that the differences among the 24 Chinese animals were small. The genetic distance between the Qinghai Tibetan pig and the Chinese northeast wild boar was the largest (0.007), and between the Yimenghei pig was only 0.001, Chinese northeast wild boar forms a distinct branch. European animals form a distinct outgroups.

**Figure 1.**
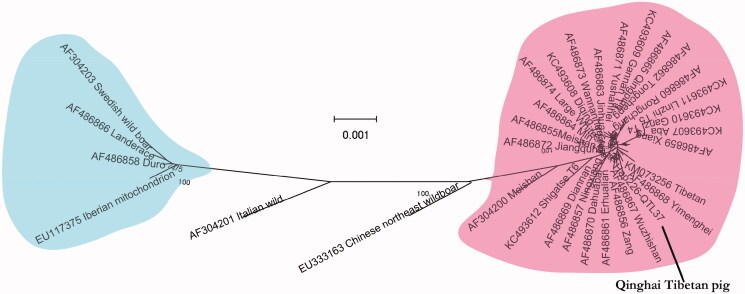
Phylogenetic trees based on the complete mitochondrial genome by Neighbor-joining analysis

The evolutionary history was inferred using the Neighbor-Joining method (Saitou & Nei, 1987). The optimal tree with the sum of branch length = 0.02986597 is shown. The percentage of replicate trees in which the associated taxa clustered together in the bootstrap test (1000 replicates) are shown next to the branches (Felsenstein, 1985). The tree is drawn to scale, with branch lengths in the same units as those of the evolutionary distances used to infer the phylogenetic tree. The evolutionary distances were computed using the Kimura 2-parameter method (Kimura, 1980) and are in the units of the number of base substitutions per site. The analysis involved 33 nucleotide sequences (32 sequences from GenBank including: KC493607-12, AF486855-73, AF304200-3, KM073256, EU333163 and EU117375). All positions containing gaps and missing data were eliminated. There were a total of 15973 positions in the final dataset. Evolutionary analyses were conducted in MEGA7 (Kumar et al., 2016).
